# Oneness, Complexity, and the Distribution of Disease

**DOI:** 10.3201/eid1109.AC1109

**Published:** 2005-09

**Authors:** Polyxeni Potter

**Affiliations:** * Centers for Disease Control and Prevention, Atlanta, Georgia, USA

**Keywords:** Art and science, emerging infectious diseases, Jackson Pollock, Autumn Rhythm, New York School, abstract expressionism, HIV, malaria

**Figure:**
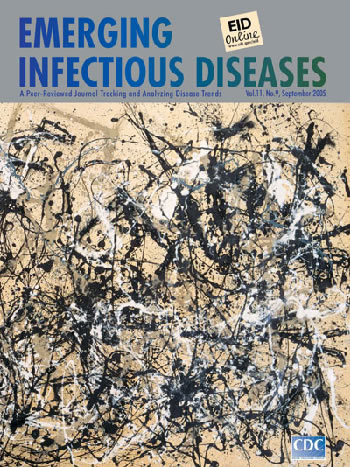
Jackson Pollock (1912–1956). Autumn Rhythm (Number 30) (1950) Enamel on canvas (266.7 cm × 525.8 cm). The Metropolitan Museum of Art, George A. Hearn Fund, 1957 (57.92) Photograph copyright 1998 The Metropolitan Museum of Art

"On the floor," said Jackson Pollock, "I feel more at ease. I feel nearer, more a part of the painting… I can walk around it, work from the four sides and literally be in the painting…. akin to the… Indian sand painters of the West" (*1*). Immediacy to the art work is hallmark of abstract expressionism or the New York School, a revolutionary art movement that shifted the center of artistic avant-garde from Europe to the United States. Born in the aftermath of World War II, this movement valued the inner world over external objects and articulated an emotional landscape fraught with uncertainty and despair.

"Modern art to me is nothing more than the expression of contemporary aims of the age that we're living in," Pollock wrote (*1*). The age was the postwar period, filled with anxiety, shocked by the atom bomb, changed by machines, immersed in Freudian theory and psychoanalysis, swinging with improvisational jazz. To this age, Pollock brought abstraction suited to his radical prototype, volatile personality, roughness, and impatience. Along with Willem De Kooning, Barnett Newman, Mark Rothko, and others, he elevated the act of painting, advocating that it should be as direct and fundamental as what it was trying to express and that it could, itself, promote emotional expression.

"I continue to get further away from the usual painter's tools such as easel, palette, brushes…. I prefer sticks, trowels, knives and dripping fluid paint or a heavy impasto with sand, broken glass…" (*1*), said Pollock about his expressive technique, which came to be known as action painting. The need to make an original statement, always at the heart of artistic and other human endeavor, permeated not only the subject matter of abstract expressionism but also its technical execution. Well-versed in the language of art, he knew and admired the work of Picasso, the surrealists, and Mexican muralists and experimented with several styles, seeking a new idiom.

"I shall be an Artist of some kind. If nothing else I shall always study the Arts…," wrote Pollock as a young man (*2*). He was born in Cody, Wyoming, the youngest of 5 children and migrated with his family to Arizona and California, where he studied art at Manual Arts High School in Los Angeles. At 18, he moved to New York City, settled in Greenwich Village, and enrolled in the Art Students League, where he studied with American Regionalist painter Thomas Hart Benton, later his mentor and friend.

"Thank you, but not so hard, not so hard," he admonished a colleague who hit him in frustration (*2*). Pollock was known to use his artist's hands in anger. He was excitable, contentious, and mistrustful of authority. He got in fights and more than once slugged his instructors in school. Those who knew him in his early years thought he could not draw. As an adult, he struggled with creative blocks, depression, and alcoholism and was torn with self-doubt. Two of his brothers, who lived with him in Manhattan, encouraged him to seek psychoanalysis, thinking that if he could "hold himself together," his work would become "of real significance," because his painting was "abstract, intense, evocative…" (*3*). Jungian psychoanalysis, particularly the conflict between reason and the unconscious, made Pollock aware of how central his emotions had become in his life and work (*4*).

In 1943, Pollock's work attracted the attention of Peggy Guggenheim, influential art dealer and patron, who commissioned a large mural. He tore down the walls of his Greenwich apartment to accommodate the huge canvas and completed the painting in a marathon 15 hours. In 1945, he left the city for East Hampton, Long Island. There in the countryside and away from distractions, he did his most innovative work. He died at age 44 of injuries in a car wreck, not knowing what he had accomplished or could have accomplished in a full life span.

"The modern artist is working with space and time and expressing his feelings rather than illustrating," Pollock believed (*1*). For hours he sat on the back porch of his farm house, taking in the natural environment, absorbing its shapes and complexity, which would later find their way into his thick interwoven designs. His studio, a converted barn, allowed space for large yachting canvases he bought at a nearby hardware store (*5*). He invented a new way to apply paint, one that combined maximum spontaneity with rigid control and produced images unprecedented in the history of art. "Dancing" on the spread canvas, Pollock created on its surface spontaneous images shaped by the trajectory of his motions. He controlled, adjusted, and modified color and shapes in paintings that were continuous, complex, and provocative and contained none of the traditional elements of composition—perspective, balance, borders, beginning, end.

"Is this a painting? Is this a painting?" he agonized (*6*). Not alone in his bewilderment, the artist was torn between marveling at and doubting his creation. "Jack the dripper," he was dubbed by the critics, who knew not what to make of these awesome tangles of paint. Was he making a profound statement or "flinging paint in the public's face?" (*7*).

Autumn Rhythm, on this month's cover, is characteristic of Pollock's most mature work. The painting contains no illusion of physical space or shapes, only textured color and seemingly unrelated lines. Reaction can only come from what the viewer feels or sees in the painting, from the striations of color and texture, which come to life as the eye moves from web cluster to color mass, discovering depth in flat surfaces, relationships in unconnected lines, muted softness, deep darkness, a delicate weave of lines, dots, swirls, and curves, overlapping into a labyrinth of infinite possibilities.

"I am nature," Pollock asserted (*6*). Through his creative "dance" he saw himself as one, not only with the painting but with its subject as well. By pouring a constant stream of paint directly out of a can, he produced a continuous trajectory, a web of crisscrossing trajectories, filled with energy and motion and reminiscent of organic shapes, trees, woods, designs of increasing complexity—nature's fingerprint as seen from his back porch in East Hampton.

Viewing Autumn Rhythm is a personal experience, one shared with Pollock in his moment of inspiration. Caught in the colorful web, we follow the infinite enamel skeins until the complex interconnections levitate off the canvas and we become part of the web.

The oneness and complexity of Pollock's paintings, his spontaneous dance to the rhythm of nature and the riddles of the inner world, speak to the biomedical scientist in a direct and fundamental way the artist would have applauded. For, in science as in art, entangled trails lead to unanticipated discoveries.

Disease distribution follows the complex, repetitive, and cumulative patterns of nature. Like Pollock's creations, it traverses spontaneous routes and arrives at unpredictable destinations. In some infectious diseases, malaria for one, incidence and spread have been defined by environmental factors: rainfall, temperature, elevation, and distribution of vector mosquitoes. Emergence of HIV unveiled additional interconnections as it inflated immunocompromised populations. No longer defined by vector distribution alone but now linked to distribution of HIV, malaria cases and deaths have increased (*8*), turning an old scourge and a new one into unlikely partners.
